# Mating system induced lags in rates of range expansion for different simulated mating systems and dispersal strategies: a modelling study

**DOI:** 10.1007/s00442-023-05492-w

**Published:** 2024-01-03

**Authors:** W. H. Morgan, S. C. F. Palmer, X. Lambin

**Affiliations:** School of Biological Sciences, University of Aberdeen, Zoology Building, Tillydrone Avenue, Aberdeen, AB24 2TZ UK

**Keywords:** Environmental change, RangeShifter, Individual-based model, Metapopulation, Informed dispersal

## Abstract

Mismatches between current and potential species distributions are commonplace due to lags in the response of populations to changing environmental conditions. The prevailing mating system may contribute to such lags where it leads to mating failure at the range edge, but how active dispersers might mitigate these lags using social information to inform dispersal strategies warrants greater exploration. We used an individual-based model to explore how different mating systems for species that actively search for habitat can impose a filter on the ability to colonise empty, fragmented landscapes, and explored how using social information during dispersal can mitigate the lags caused by more constrained mating systems. The mate-finding requirements implemented in two-sex models consistently led to slower range expansion compared to those that were not mate limited (i.e., female only models), even when mating was polygynous. A mate-search settlement strategy reduced the proportion of unmated females at the range edge but had little impact on rate of spread. In contrast, a negative density-dependent settlement strategy resulted in much faster spread, which could be explained by a greater number of long-distance dispersal events. Our findings suggest that even low rates of mating failure at the range edge can lead to considerable lags in range expansion, though dispersal strategies that favour colonising more distant, sparsely occupied habitat patches may effectively mitigate these lags.

**Supplementary Information:**

The online version contains supplementary material available at 10.1007/s00442-023-05492-w.

## Introduction

Predicting how species ranges will shift in response to environmental change is one of the key contemporary challenges in ecology (Parmesan 2006; Maguire et al. 2015). The ability of species to keep pace with rapid change over the time scale of tens of generations or fewer will be influenced by a range of life history traits, including dispersal and fecundity, as well as by their mating strategy and the breadth of tolerance to environmental conditions (Buckley and Kingsolver 2012; Macgregor et al. 2019). Together, these traits determine (1) where persistence is possible, (2) where colonisation is possible, and (3) the length of any lag before colonisation occurs (Alexander et al. 2018). An inability of populations to spread into newly suitable habitat as it becomes available can result in what have been termed ‘colonization credits’, in analogy to the better known concept of extinction debt (e.g., Talluto et al. 2017). This effect, coupled with a rapid decline in occupancy of regions that become unsuitable, can result in substantial reductions in a species’ range size (Rumpf et al. 2019). Furthermore, species with lower capacity for spread will lag behind their more mobile counterparts, with consequences for community composition and functioning in newly emerging ecosystems (Urban et al. 2012).

In fragmented landscapes it is useful to think in terms of a “metapopulation persistence threshold”, above which sufficient, accessible breeding habitat of a high enough quality exists for the colonisation and eventual long-term persistence of a particular species, given the baseline rate of local extinction (Hanski and Ovaskainen 2000). Where the availability or quality of suitable habitat changes along a gradient (e.g., altitudinal or latitudinal), the persistence threshold will determine the eventual location of the range boundary, and in time, a state of equilibrium with the landscape may be reached (Holt and Keitt 2000). There is a growing appreciation of the fact that ranges are often not at equilibrium and many species currently occupy a smaller geographical distribution than the abiotic environment and biotic conditions might allow (García–Valdés et al. 2013; Talluto et al. 2017); their realised versus fundamental niche (Hutchinson 1957). Species with slow demography or limited dispersal ability may be particularly prone to lags in their response to changing environmental conditions (Alexander et al. 2018). For instance, García–Valdés et al. (2013) predicted that nine of the 10 most common tree species of mainland Spain would remain out of equilibrium with current climatic conditions until at least the end of the twenty-first century. Lags of this length could have significant impacts on species’ geographical distributions under rapid environmental change, where temperature isoclines have been predicted to move poleward at 2–8 km per year (Schippers et al. 2011).

Expansions of populations through landscapes containing suitable habitat act like travelling waves (Birzu et al. 2018), where the range edge can be “pulled” by high growth rates at the front or “pushed” due to demographic momentum from behind (Neubert et al. 2000; Walter et al. 2015). When local growth rates are depressed at low densities (an Allee effect; Stephens et al. 1999), range fronts tend to be pushed forward by dispersing individuals from higher density areas spilling over the edge and colonising previously unoccupied habitat patches (Kot et al. 1996). Infrequent encounters between opposite-sex conspecifics at the range edge (a mate finding Allee effect; Gascoigne et al. 2009) can cause depressed growth rates and may, therefore, lead to a pushed front with long lags before onward range expansion (Contarini et al. 2009; Tobin et al. 2009; Shaw and Kokko 2015).

The prevailing dispersal strategies of a species may contribute to the prevalence of substantial lags in the colonisation of newly suitable parts of the landscape. For actively dispersing species, predicting the rate of expansion through fragmented landscapes is challenging due to the often complex interactions between dispersal strategies and features of the landscape (e.g., proportion of suitable habitat: Bocedi et al. 2014b). Such strategies often involve the use of social information (sensu Danchin et al. 2004), attraction to conspecifics, avoidance of crowded patches and searching for potential mates, which may all affect expansion by influencing the rate at which dispersing individuals select newly suitable and unoccupied habitat patches (Clobert et al. 2009).

Dispersal strategies appear to be intrinsically linked to species’ mating systems, though the degree to which inbreeding (or selfing) reduces fitness is likely important in determining whether inbreeding avoidance is positively or negatively associated with dispersal ability (Cheptou 2012; Auld and Rubio de Casas 2013). While the combination of long distance dispersal and selfing may be particularly advantageous for rapid colonisation (Baker 1955, 1967), limited attention has been paid to understanding the coevolution of dispersal and mating systems, particularly during range expansion (Hargreaves and Eckert 2014; but see Iritani and Cheptou 2017). For sexually reproducing populations, combinations of traits relating to both dispersal and mating strategy (i.e., a dispersal-mating strategy) are likely to influence lags in range expansion (Shaw and Kokko 2015; Shaw et al. 2017). Simulations suggest that the need to find a mate in the landscape can lead to reduced female mating rate and several-fold increases in the number of generations required to spread a given distance, and such lags are further accentuated when mating is obligate monogamous (Shaw and Kokko 2015). Species that opt for, or that are constrained by, monogamy (e.g., through reliance on biparental care) might, therefore, suffer the most acute consequences of rapid environmental change.

Actively searching for potential mates within suitable habitats before settling may increase the chance of metapopulation persistence and rate of expansion if this searching behaviour leads to more frequent encounters between opposite-sex conspecifics (i.e., by the avoiding of single-sex patches) than if individuals settle in the first suitable habitat patch they find (South and Kenward 2001; Shaw et al. 2017). Similarly, polygynous mating (one male mating with multiple females) should also reduce the number of females who remain unmated, thereby leading to faster rates of expansion (Shaw and Kokko 2015; Shaw et al. 2017). Simulation models have also suggested that avoiding high density patches may increase the rate at which species colonise suitable habitat (Bocedi et al. 2014b; Stodola and Ward 2017). A suggested mechanism for this is that mates become easier to find as negative density-dependent settlement causes a greater abundance of dispersers to reach the range front (Bocedi et al. 2014b). Accordingly, a developed understanding of the effect of mate-searching and negative density-dependent settlement on mating success at the range edge, and how this influences range dynamics, will help to improve predictions of spread under environmental change further.

Here, we use an individual-based model (IBM) to explore how mating systems for species that can actively search for habitat can impose a filter on the ability to colonise empty, fragmented landscapes for species having particular trait sets. We test how mate limitation for populations with monogamous and polygynous mating systems affects the speed of range expansion across a range of habitat qualities, fecundities and dispersal behaviours. We then explore spatial dependencies of female mating success and emergent dispersal distances under different dispersal-mating strategies.

## Methods

We used the spatially explicit, individual-based modelling platform RangeShifter (Bocedi et al. 2014a) to simulate metapopulation dynamics and spread of a single species in a landscape of suitable habitat patches within an unsuitable matrix. We used RangeShifter v2.0 (Bocedi et al. 2021), which enables the initialisation of specified patches with specified numbers of each sex. We did not set out with the aim of mimicking all the complexities of any real-world system but sought to include enough realism with which to explore the processes of interest. In particular, we took inspiration from some metapopulation systems where dispersal between, and settlement within, habitat patches is relatively unambiguous (Lambin et al. 2004).

**Fig. 1:**
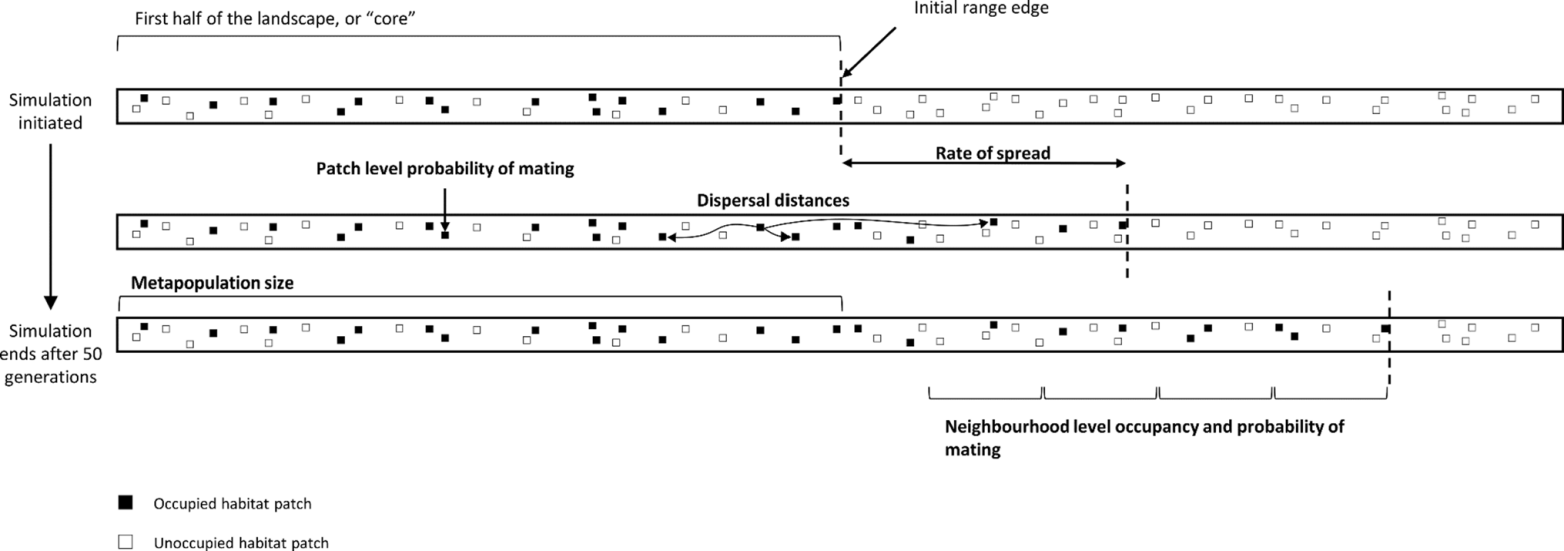
A conceptual diagram showing the corridor-like landscape in which all simulations took place and the core of the landscape that was populated at the start of each simulation. Vertical dashed lines show the range edge at each time point. Emboldened text denotes metrics that were monitored during all simulations

All simulations were carried out in a single corridor-like gridded landscape with hard borders (i.e., non-periodic boundary conditions) of 100 m × 100 m cells (20 columns × 1000 rows). This configuration was chosen to simplify the process of monitoring the location of the range edge (see below for more details). Seven percent of cells were randomly selected as suitable breeding habitat, each of which constituted a separate habitat patch (Fig. 1). This value was chosen as it is similar to the proportion of suitable habitat in a well-studied small mammal metapopulation (Sutherland et al. 2014). The remaining 93% of cells were classified as unsuitable matrix through which dispersal could occur. For all simulations, half of the suitable cells in the first 500 rows of the landscape were populated. The initial number of individuals per patch was sampled from a zero truncated Poisson distribution with mean *λ* = 2. For two-sex models, there were independent draws for each sex in each patch.

We used a stage-structured model with overlapping generations, in which different vital rates were assigned to juveniles (individuals that are < 1 year old) and adults (those ≥ 1 year old). Individuals could live for a maximum of 3 years and adults could produce offspring once each year. These models are, therefore, particularly relevant for many short-lived species, including numerous small mammal, bird and amphibian species (de Magalhaes et al. 2005), and some insects, including butterflies.

### Mating systems

To characterise metapopulation dynamics and spread when populations are not limited by mate-finding ability (i.e., when males can find and mate with all females), we used a female-only model. To explore the effect of more constrained life histories, we used a two-sex model with two different mating systems: (1) males and females must occupy the same patch to mate, and males can mate with multiple females (polygynous mating), and (2) males and females must occupy the same patch to mate, and males can only mate with one female (obligate monogamous mating). To generate these mating systems, we constrained the maximum number of mates a male could have to 100 (polygynous mating) or 1 (monogamous mating) respectively, which we modelled as follows (Bessa–Gomes et al. 2010):

1 $$c=min\left(1,\frac{2hm}{f+hm}\right) f$$

where $f$ and $m$ are the numbers of potentially reproductive females and males in a patch, respectively, and $h$ is the harem size, i.e., the maximum number of females with which a male could mate. Each potentially reproductive female had a probability of reproducing, $Pr$, given by:

2 $$Pr=\frac{c}{f}$$

A Bernoulli trial, $Bern\left(Pr\right)$, determined if the female reproduced or not.

Adult females produced offspring at the start of the year with a probability of 1 if there was a male available to mate with. The number of offspring produced per female was density independent and was drawn from a Poisson distribution with mean equal to adult female fecundity (see Experiment 1, below). For a comparable two-sex model, fecundity was doubled and an individual’s probability of either sex at birth set to 0.5. See supplementary materials for examples of RangeShifter parameter files for different models.

### Dispersal

Offspring then emigrated with a fixed probability (Table 1) or remained in their natal patch and, therefore, models with higher fecundity produced more emigrants per habitat patch. Only the juvenile life stage could emigrate. Dispersal movement was modelled using the stochastic movement simulator (SMS; Palmer et al. 2011), which is embedded in RangeShifter. SMS simulates discrete, step-wise movements through the landscape, where an individual’s perceptual range (PR), its tendency to follow a correlated trajectory (directional persistence, DP) and the relative cost of moving through habitat or non-habitat cells, contribute to the probability of an individual moving into a neighbouring cell (parameters in Table 1). We imposed no maximum number of steps that an individual could take, such that dispersal distances emerged as a result of the spatial configuration of suitable habitat cells, settlement rules, and per-step probability of mortality (Table 1).

**Table 1:** RangeShifter parameters that were held constant throughout all simulations

Model components	Parameters
Demography	
Adult survival probability	0.3
Dispersal	
Emigration probability	0.5
Perceptual range (cells)	5
Directional persistence	3.0
Per step mortality rate	0.005
Relative movement cost	
Habitat	1
Matrix	10

Directional persistence corresponds to the tendency of an individual to follow an autocorrelated trajectory. A value of 3.0 means an individual is 3.0^2^ or 3.0^4^ times less likely to make a 90° or 180° turn, respectively, than continue in a straight line

### Survival

After dispersers immigrated into a new patch, stage-specific survival probability was applied to all individuals. We modelled stage-specific density-dependent survival probability weighted such that only females contributed to the calculation of survival probability:

3 $${\sigma }_{i,j,t}={\sigma }_{0,i}*{e}^{-{\text{b}}{N}_{f,j,t}}$$

where σ_i,j,t_ is the survival probability of stage *i* in patch *j* at time *t*, ${\sigma }_{0,i}$ is the maximum survival probability of stage *i* at low density, *N*_*f,j,t*_ is the total number of females in patch *j* at time *t,* and *1/b* is the strength of density dependence. This weighting by sex of the contribution to density dependence allowed us to make direct comparisons between female only and two-sex models, i.e., males only contributed fertilisation and did not affect demographic rates. Given that all habitat patches in the landscape were of equal size (1 ha), and only the number of females on the patch affected local survival probability, we considered the strength of density dependence (1/b) on survival (in units of females/patch) to describe habitat quality of suitable patches in the landscape.

### Experiment 1—Effect of mating systems on metapopulation size and spread

To assess how mating systems influence (1) metapopulation size within and (2) spread through newly available landscapes, we varied habitat quality (1/*b* = females/patch), mating system and fecundity in a fully factorial design (Table 2) and kept all other parameters constant (Table 1). Simulations ran for 50 generations, and we carried out 5 replicates per parameter combination.

**Table 2:** Parameters varied in a fully factorial design to test the effect of mating system on metapopulation size and spread through a fragmented landscape

Mating system	Habit quality		Fecundity	
(harem size, *h*)		1/*b*		Mean offspring/female
Female only	x	2	x	2.0
Two sex (1)		4		3.0
Two sex (100)		6		4.0
		8		
		10		
		12		
		14		

Harem size is the maximum number of females a male can mate with, and 1/b = females/patch

### Metapopulation size

Metapopulation size was measured using proportional occupancy in the first half (i.e., the first 500 rows) of the landscape (which we refer to as the “core” hereafter) after 50 generations, where an occupied patch was defined as one with a population capable of producing offspring (Fig. 1). To reveal which factors were most important in determining metapopulation size, we fitted a linear model that included all main effects (habitat quality, fecundity and mating system) and two-way interactions. Linear models were used as the main objective of the statistical analysis was to reveal which terms explained the greatest amount of variance in the dependent variables (sensu Barros et al. 2016). We used the R package “relaimpo” and the calc.relimp function (using type = “lmg”) to calculate the amount of variance explained by each main effect and interaction (Grömping 2006). Following this, we determined which populations were able to maintain quasi-equilibrium occupancy in the landscape. For models that did not go extinct before year 50, we derived the yearly change in proportional occupancy (Δ*p*) in the core between years 20 and 50 of the simulations, and those populations where average Δ*p* + 1 standard error (SE) > 0 were considered to be able to maintain quasi-equilibrium occupancy.

### Rate of spread

To assess how habitat quality, mating system and fecundity affected rate of spread, we monitored the number of extra rows colonised every five generations and divided this by five. This gave an overall rate of spread (rows/generation; Fig. 1). The range edge was defined as the furthest forward habitat patch with a breeding population (Fig. 1). Again, we fitted a linear model including all main effects and two-way interactions.

### Probability of mating

To establish what influence the mating system had on the proportion of females that did not reproduce, we inferred the number of unmated females on each patch based on the mating system and the harem size, i.e., for polygynous models, for all females to mate only one male was required, but a 1:1 ratio was required in monogamous models. We fitted a generalised linear model (GLM: binomial error structure, logit link function) to patch-level proportions of unmated females as a function of the interaction between mating system and patch-level population size. We controlled for fecundity by including it as a main effect in the model. We did not include female-only simulations in this statistical model, where no female remained unmated by design.

### Experiment 2—Dispersal strategies

#### Rate of spread

To explore how dispersal strategies can modify a species’ ability to exploit habitats of differing quality, we used a two-sex model with either a polygynous or monogamous mating system. We compared the following dispersal strategies: (1) Habitat only: a disperser settled whenever it found a suitable habitat patch; (2) Mate-search: a disperser settled only if there was at least one opposite sex conspecific in the patch (where the patch was empty in the previous year, both individuals had to arrive simultaneously); (3) negative density-dependent settlement, where individuals avoid settling in more densely population patches; and 4) Mate-search and density-dependent settlement. For density-dependent settlement, individuals had a probability, ${P}_{s}$_*,*_ of settling in patch *i*, given by:

4 $${P}_{s}=\frac{1}{1+{e}^{-\left(b{N}_{i}-{\beta }_{s}\right)*{\alpha }_{s}}}$$

where again, *b* represents strength of density dependence, *N*_*i*_ is the number of females in cell *i*, *β*_*s*_ is the inflection point and* α*_*s*_ is the slope of the function. We carried out a fully factorial design of all settlement strategies and four values of habitat quality (1/b = 8, 10, 12 and 14 females/patch; a subset of those used in experiment 1, and values at which all models could maintain quasi-equilibrium occupancy; Figure S1 supplementary materials) and held mean fecundity constant at 3.0 offspring/female. For all scenarios with density-dependent settlement we used *β*_*s*_ = 0.75 and *α*_*s*_ = − 10. Again, we ran simulations for 50 years, with 5 replicates per parameter combination. We assessed the impact of these dispersal strategies on rate of spread, which was calculated as above. In some models the whole landscape was colonised before the end of the simulation, therefore only the first 30 years were used to calculate spread rates.

#### Probability of mating

To explain why differences in the rate of spread might emerge from different dispersal-mating strategies, we evaluated how the proportion of unmated females varied with “neighbourhood occupancy”. A neighbourhood was defined as all habitat patches within a 10-row section of the landscape, and each year the occupied range was split afresh into these neighbourhoods starting from the range edge, such that the relative location of each neighbourhood in the occupied range remained constant (Fig. 1). We monitored proportional occupancy and proportion of unmated females in every neighbourhood each year of the simulation.

#### Dispersal distances

To further explain differences in the rate of spread, we evaluated how mate-search and density-dependent settlement rules influenced the emergent dispersal kernels for simulated populations. We evaluated the number of rows dispersed by females that successfully completed dispersal (Fig. 1) for a single value of habitat quality (1/b = 10) and mean fecundity (3.0 offspring/female).

## Results

### Experiment 1—Effect of mating systems on metapopulation size and spread

**Fig. 2:**
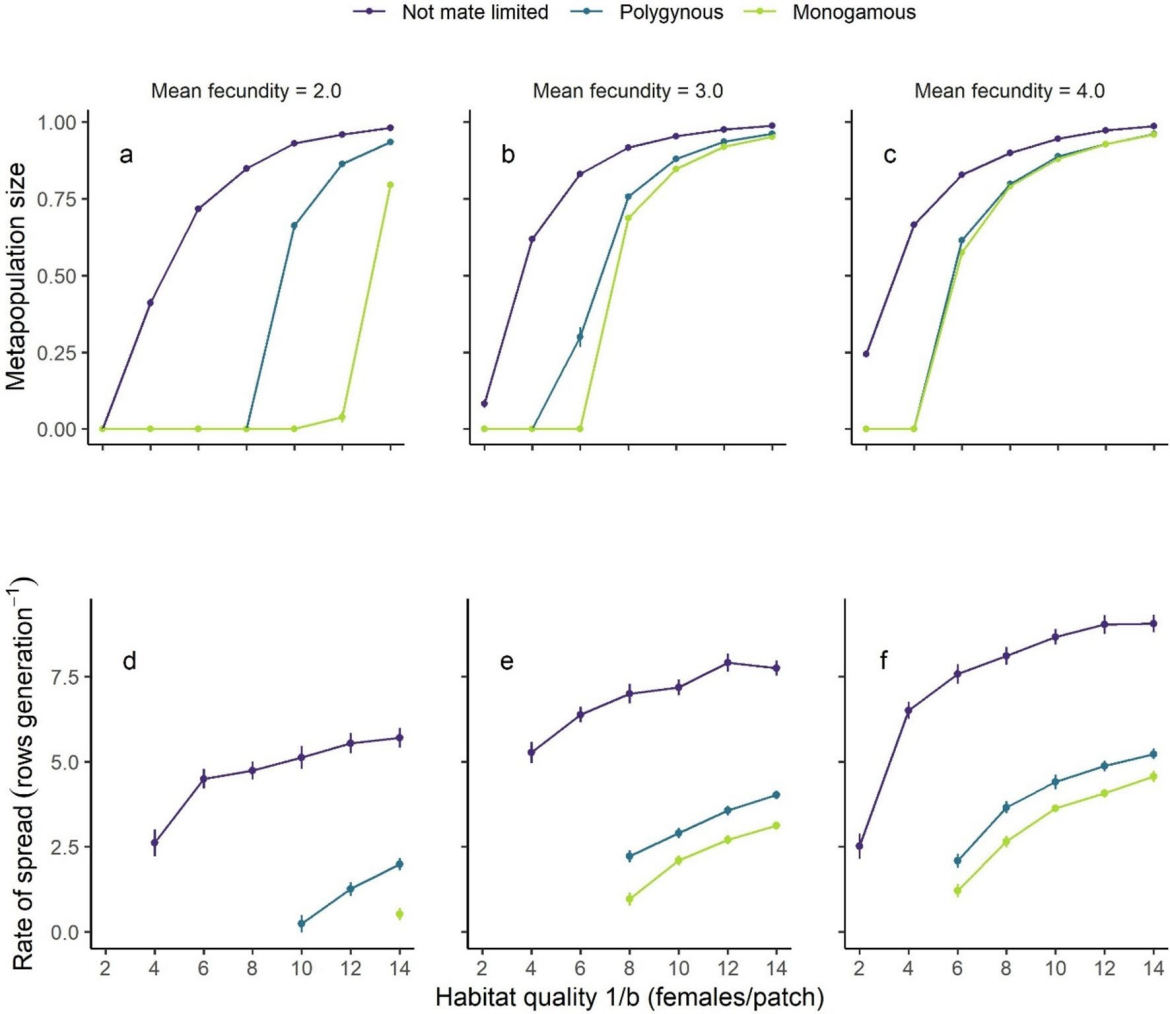
The effect of habitat quality (1/b) on **a**–**c**) metapopulation size and **d**–**f**) rate of range expansion under three mating systems for three values of fecundity (mean offspring/female). Error bars show the standard error. Rate of spread is only shown for models where the species could maintain quasi-equilibrium proportional occupancy

We found large variation in metapopulation size and rate of spread due to mating system, though mate-finding requirements (i.e., two-sex simulations where male dispersal was modelled explicitly) consistently led to smaller metapopulations (Fig. 2a–c), and slower rates of spread (Fig. 2d–f).

### Metapopulation size

Metapopulation size in the core was driven primarily by habitat quality, which explained 60% of the variance (Table 3). In contrast, fecundity and mating system explained only 8% and 13% of the variance in metapopulation size, respectively, and all two-way interactions explained a further 13%. Decreasing habitat quality led to reduced metapopulation size across all mating systems (Fig. 2a–c), and models with mate-finding requirements and low fecundity in landscapes with low habitat quality had the smallest metapopulation sizes and declined to extinction most often. Models with no mate-finding requirements (i.e., female only models) went extinct in only 5% of simulations, whereas 38% of polygynous model simulations and 47% of monogamous ones went extinct within 50 years. Only simulations where the species could maintain quasi-equilibrium in proportional occupancy were included in further analyses (Figure S1 supplementary materials).

**Table 3:** Variance (%) in metapopulation size (measured as proportional occupancy in the core) and rate of spread that was explained by habitat quality (HQ), mean fecundity (F) and mating system (M)

Response	Effect	Df	Sum squares	Mean square	Variance explained
Proportional occupancy	HQ	6	31.8	5.3	59.5
	F	2	4.3	2.2	8.1
	M	2	6.9	3.4	12.9
	HQ:F	12	2.5	0.2	4.7
	HQ:M	12	3.0	0.3	5.7
	F:M	4	1.4	0.3	2.5
Rate of spread (rows/generation)	HQ	3	408.6	136.2	4.3
	F	1	568.3	568.3	6.0
	M	2	5975.4	2987.7	63.1
	HQ:F	3	2.5	0.9	< 0.1
	HQ:M	6	37.7	6.3	0.4
	F:M	2	3.1	1.6	< 0.1

*Df* degrees of freedom

### Rate of spread

Variation in rate of spread was driven primarily by mating system. On average, polygynous and monogamous models spread at 48% and 40% the rate of those that were not mate limited, respectively, which equated to a lag of 3.3 and 3.8 rows/generation (Fig. 2d–f). While mating system explained 63% of the variance (Table 3), habitat quality and fecundity explained only 4% and 6% of the variance respectively, and less than 1% was explained by all two-way interactions. To avoid singularity errors during model fitting, only those parameter combinations where spread occurred for all mating systems were included (habitat quality (1/b) > 6 and mean fecundity > 2.0).

### Probability of mating

**Fig. 3:**
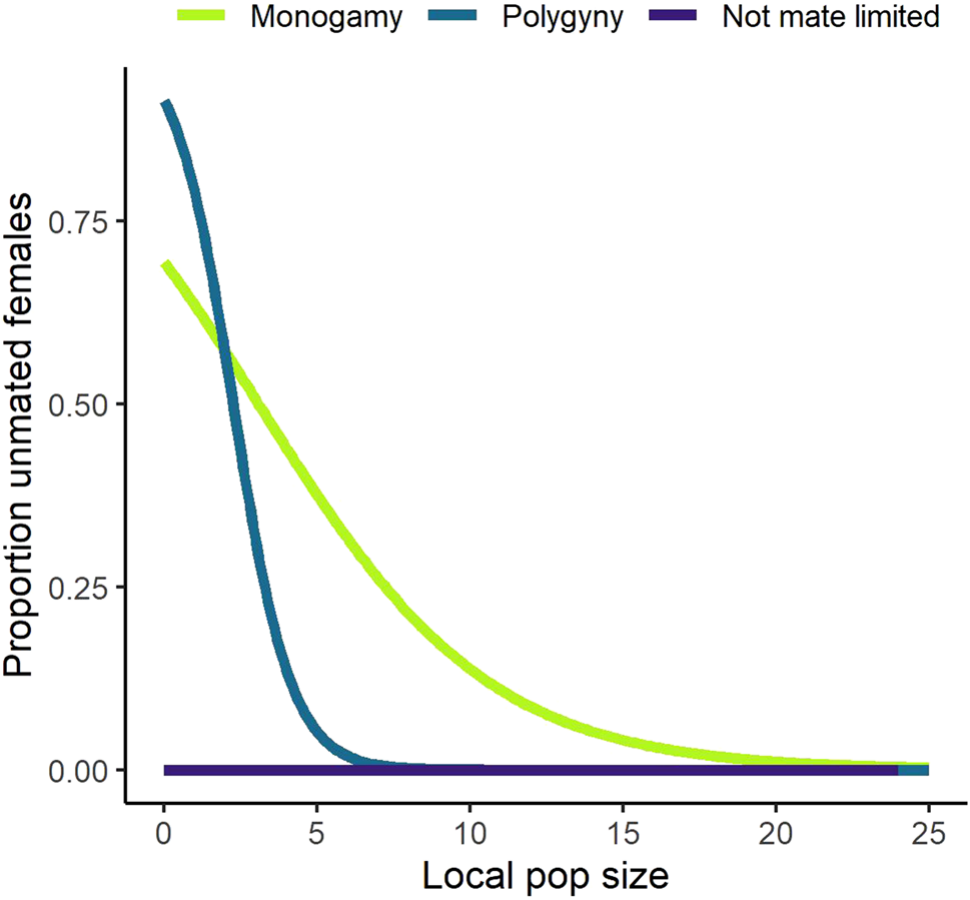
The proportion of unmated females in a patch as a function of local population size for the three mating systems, where mean fecundity = 3.0 offspring/female

Variation in rate of spread reflected variation in the realised proportion of unmated females. Mate-finding requirements caused an increase in unmated females with declining local population size, and this effect was most dramatic for monogamous populations (Fig. 3).

### Experiment 2—Dispersal strategies

#### Dispersal-mating strategies and rate of spread

**Fig. 4:**
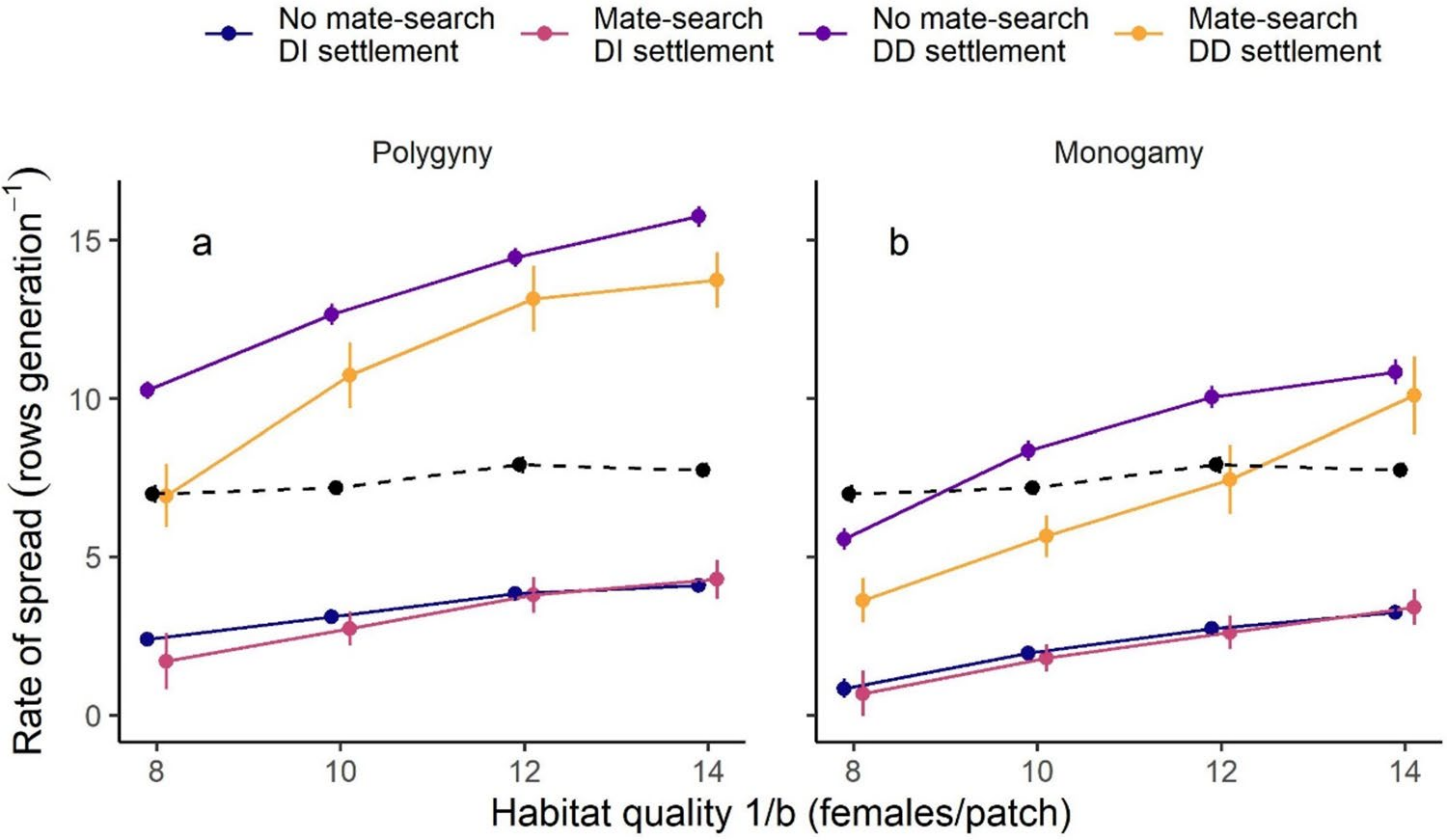
The effect of alternative dispersal strategies on the rate of range expansion for models with **a** polygynous and **b** monogamous mating systems. *DI* density independent, *DD* density dependent. The dashed lines show the rate of expansion for a female-only model not limited by mate finding requirements (but uninformed dispersal, i.e., density independent settlement only)—this is replicated on both panels for comparison. Error bars show the standard error, and points have been jittered on the x-axis to show overlapping error bars

Density-dependent settlement led to faster rates of spread, and when habitat quality (1/b) was > 6 females/patch for polygynous models and > 8 females/patch for monogamous models this fully mitigated the lags in expansion caused by mate-finding requirements (Fig. 4). Mate searching alone did not affect the rate of range expansion when compared to models with habitat only settlement rules. However, models with both mate searching and density-dependent settlement spread more slowly than those with only a density-dependent settlement rule (Fig. 4).

#### Dispersal-mating strategies and probability of mating

**Fig. 5:**
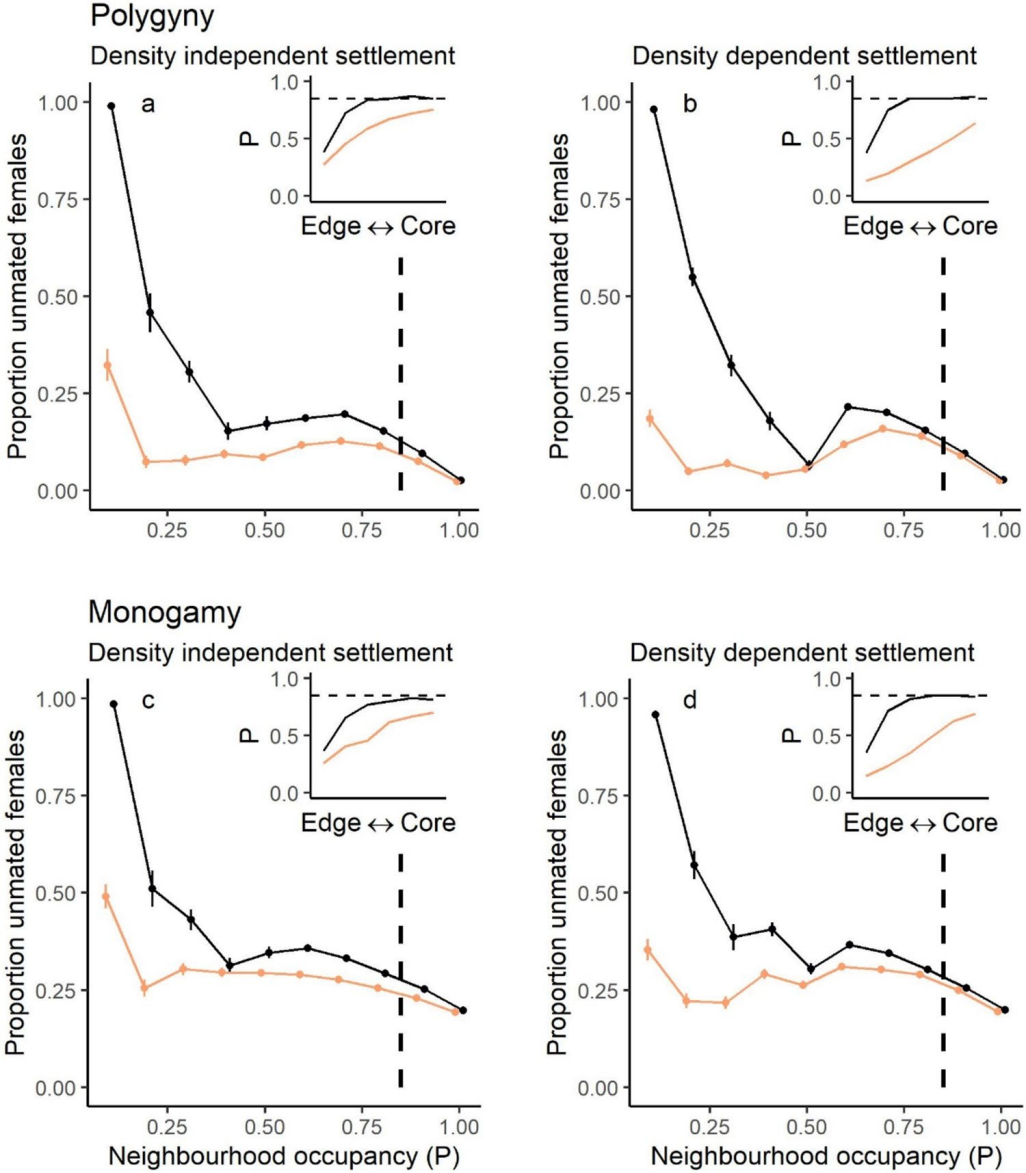
The relationship between the proportion of unmated females and neighbourhood occupancy (P), where mean fecundity = 3.0 offspring/female and habitat quality (1/b) = 10 females/patch. Black points = no mate-searching, orange points = mate-searching. The dashed lines are at *P* = 0.85, which is representative of the average proportional occupancy in the core of the range for these parameter combinations. Error bars show the standard error, and points have been jittered to show overlapping error bars. Inset plots show spatial variation in P to indicate how the proportion of unmated females declined towards the range edge. Black = no mate-searching, orange = mate-searching; the dashed line is at *P* = 0.85

The influence of the mating system and dispersal strategy on the proportion of unmated females varied according to neighbourhood occupancy (Fig. 5).

In core parts of the range, where patch occupancy was at quasi-equilibrium (neighbourhood occupancy ~ 0.85 where 1/b = 10 females/patch, mean fecundity = 3.0 offspring/female), the prevailing mating system strongly influenced the proportion of females that remained unmated, while the dispersal strategy had little impact (Fig. 5). In polygynous models around 2–15% of females remained unmated in these core areas, whereas in monogamous models around 19–30% of females failed to mate (Fig. 5).

Near the range edge (neighbourhood occupancy < 0.85 where 1/b = 10 females/patch, mean fecundity = 3.0 offspring/female), the dispersal strategy caused large differences in the proportion of unmated females for both mating systems. In models without mate searching, the proportion of unmated females increased dramatically as neighbourhood occupancy decreased (Fig. 5). Mate-searching largely prevented this decline, although at the lowest neighbourhood occupancies (< 0.2), around 19–49% of females still remained unmated (Fig. 5).

#### Dispersal distances under different strategies

**Fig. 6:**
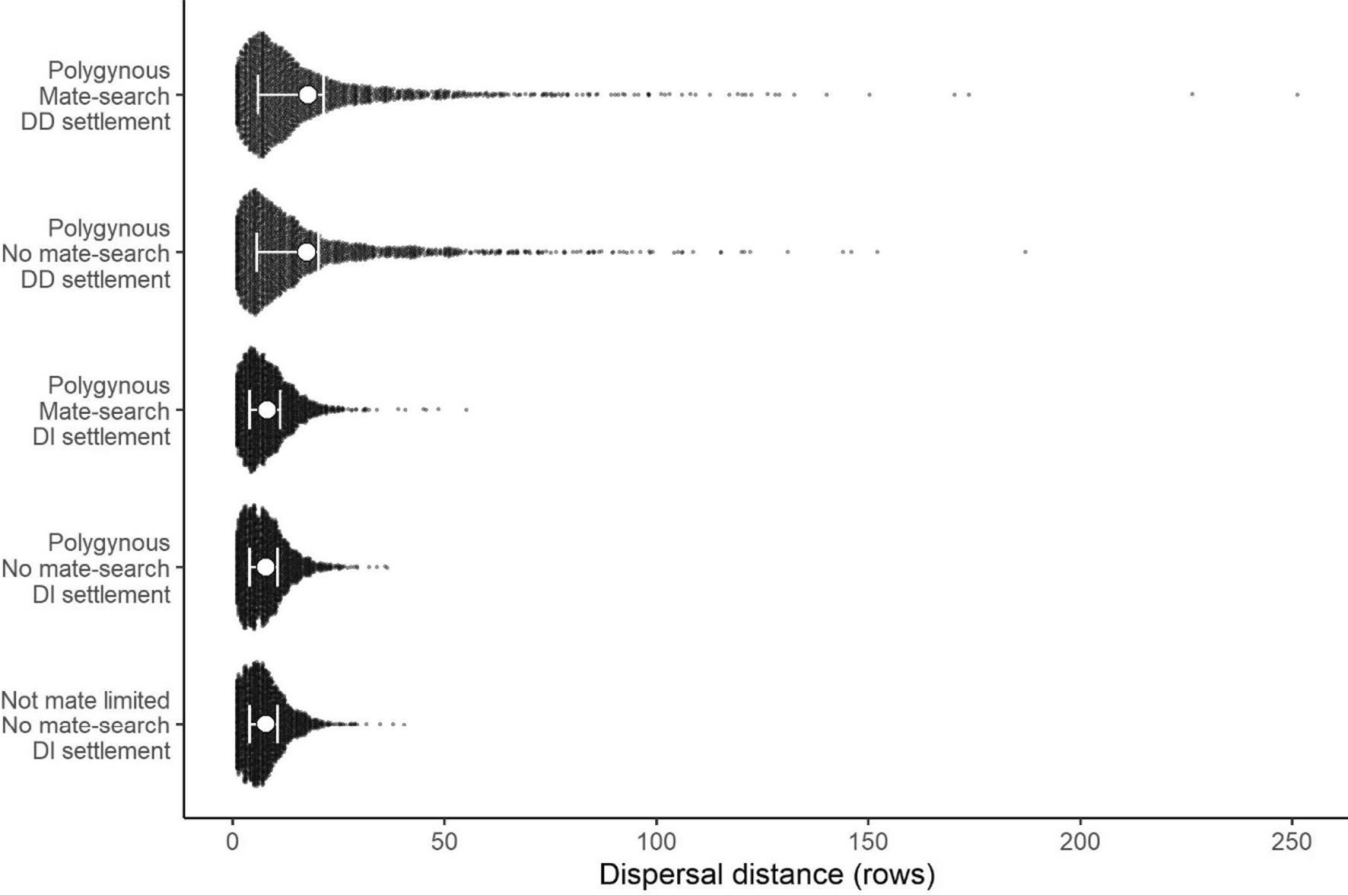
Distributions of dispersal distances for models with different settlement strategies. White points show the mean dispersal distance for that strategy, and error bars show the interquartile range. Black points show individual dispersal distances, and 2000 individuals from each strategy were randomly selected for plotting. For two-sex models with polygynous mating, only females were included, and the female-only (not mate limited) model is included for comparison

Mean dispersal distance was more than twice as long in models with a density-dependent settlement rule (mean: 17.6 rows) compared to those with a habitat only rule (8.0 rows). In contrast, mate searching had only a small impact on mean dispersal distances, leading to a 3–4% increase. Density-dependent settlement resulted in some individuals dispersing very long distances such that the dispersal kernel was long tailed (Fig. 6).

## Discussion

It is accepted that the distributions of many species will increasingly be in a state of disequilibrium as they lag behind environmental change (García–Valdés et al. 2013; Talluto et al. 2017). Here, we used simulations to show how mate-finding requirements may cause long lags in the spread of a species through fragmented landscapes over short time scales (tens of generations). Failure by individuals to find mates, particularly at the low densities arising at the range edge, was a key mechanism underlying these lags. While mate searching settlement rules successfully reduced the number of unmated females, this was not sufficient to mitigate lags in expansion. In contrast, negative density-dependent settlement rules resulted in a dramatic increase in the rate of range expansion, often exceeding rates seen in female only models with no mate-finding requirements. This could be explained by a greater number of long-distance dispersal events. Our simulations suggested that dispersal-mating strategies will play an important role in determining which species can keep up with rapid environmental change.

### Lags and mating systems

Range spread in two-sex models took place at less than half the rate as seen in models with no mate finding requirement, largely due to the increased proportion of females that remained unmated. Obligate monogamous models consistently had the slowest spread. There is much variation in the degree to which species and individuals adhere to monogamous mating strategies (Kvarnemo 2018), and this result may be most relevant for species (or populations) where individuals opt for, or are constrained by monogamy (e.g., many bird species, see below) despite having access to additional mates. One instance where this may arise is when monogamy is enforced (Hosken et al. 2009). For example, male golden orb spiders *Nephila fenestrate* may break off part of their copulatory organs during mating, protecting their paternity while sacrificing any future mating opportunities (Fromhage and Schneider 2006). Females may also enforce monogamy: female mantids *Tenodera sinesis* and *Pseudomantis albofimbriata* may cannibalise males (Hurd et al. 1994; Barry et al. 2008), and female burying beetles *Nicrophorus defodiens* can physically prevent males from attracting further mates, thereby securing greater resources and biparental care (Eggert and Sakaluk 1995). While such strategies may be beneficial for individual fitness, species that employ them may be more likely to lag behind their potential range.

One prediction arising from our simulations is that species that rely on biparental care (e.g., around 80% of birds: Cockburn 2006) may show longer lags in range expansion. This may even be the case for species that carry out extra-pair mating (i.e., polygamy) (Brouwer and Griffith 2019). While polygyny by males may ensure that all (or most) females are mated, the lack of parental care can have important consequences for female fitness. For example, polygynous male house sparrows *Passer domesticus* have been found to provide aid almost exclusively to one of their mates, such that nonaided females had reduced clutch sizes, hatching success and fledgling quality (Veiga 1990). Another pertinent example is that of hen harriers *Circus cyaneus* at the northerly edge of their range in Britain, where females have been found to outnumber males two to one (Balfour and Cadbury 1979). Balfour and Cadbury (1979) observed that more fledglings were produced by nests in monogamous situations. This highlights the complex link between behaviours that maximise individual fitness and those that may make populations vulnerable to lags in range expansion.

### Lags and mate-searching

While mate searching strategies were successful in reducing the proportion of unmated females at the range edge in both polygynous and monogamous models, this was not sufficient to influence the rate of spread when compared to settlement strategies shaped by habitat only. Simulation studies suggest that more effective mate-finding can translate into faster spread rates (Shaw et al. 2017). However, unless dispersal capacity is high enough for individuals to sequentially visit multiple patches, it seems likely that some females will always remain unmated and a mismatch between potential and realised range will emerge. For example, despite the use of pheromones by gypsy moths *Lymantria dispar* to improve mate finding, at the expanding edge of their invasive range many females remained unmated due to low male moth densities (Contarini et al. 2009; Tobin et al. 2013). This has been associated with a slowing of the rate of expansion (Tobin et al. 2007). More mobile species may have no such constraints. For example, in an invasive population of American mink *Neovison vison*, the probability of females conceiving a litter was unaffected by the density of males, despite both sexes being heavily depleted due to ongoing control efforts (Melero et al. 2015). A consequence of lower dispersal ability may be enforced monogamy at the range edge (Whiteman and Côté 2004; Kokko and Rankin 2006), and this is likely to result in particularly long lags in range expansion.

It is worth noting that under our mate-search settlement rule, dispersers had to arrive simultaneously on an empty patch as an opposite-sex conspecific to satisfy the conditions for settlement. There is some empirical evidence that on finding an empty patch, metapopulation-dwelling water voles *Arvicola amphibius* will delay onward dispersal for several days, presumably in the expectation that they will be joined on the patch by fellow dispersers (Fisher et al. 2009). This delaying settlement behaviour may improve mating success in patchy landscapes and form part of a syndrome of adaptation to metapopulation living. However, our results suggest that even a low proportion of female mating failure may translate into substantial lags when compared to scenarios where all females mate successfully.

### Lags and density-dependent settlement

In contrast to mate-search, a negative density-dependent settlement rule resulted in much faster rates of spread, which at higher habitat qualities even exceeded those seen in models with no mate-finding requirements. Faster spread reflected changes in the emergent dispersal kernels, with density-dependent settlement leading to a much greater prevalence of long-distance dispersal events. Under density-independent settlement rules, individuals settled in the first patch they found. Previous simulation studies have shown that this may cause a “shadow effect” where patches closer to the core of the range “block the path” to more distant patches (Hein et al. 2004), potentially constraining the rate of spread through patchy landscapes (Bocedi et al. 2014b). This effect was reduced under the simulated density-dependent settlement rule, where dispersers rejected closer, more crowded patches and continued on in search of more distant, sparsely occupied/empty ones. While it is very likely that varying the proportion of suitable habitat in our simulated landscape would also have impacted on rates of spread (see Bocedi et al. 2014b), we expected that this effect would be additive and not interact with the mating and dispersal traits that were the focus of this study. Variation in the tendency for individuals to settle in occupied patches (“joiners”) versus empty ones (“colonizers”) has important consequences for persistence of populations in patchy landscapes (Clobert et al. 2009). Under rapid environmental change, the proportion of colonizers present at the range margins (and whether differences in dispersal reflect morphological or behavioural variation) may have important consequences for the rate of spread.

In our simulations, lags caused by mating failure could be mitigated when some individuals dispersed further to find patches that were sparsely occupied/empty of females (as only females contributed to calculation of density-dependence). Such instances of long-distance dispersal may benefit both individuals (who avoid levels of competition present in high-density patches) and populations (as individuals spread out and occupy more patches in the landscape). However, mitigating lags in expansion through investing more in dispersal may not be possible under rapid environmental change if such change makes dispersal riskier, as has been suggested for pika *Ochotona princeps* that may experience temperatures outside of their physiological tolerance when dispersing through low-elevation corridors that connect boulder taluses (Smith 1974; Stewart et al. 2017). Simulations suggest that when the cost of dispersal is high, shorter dispersal distances maximise population sizes in patchy landscapes (Delgado et al. 2014). As a result, strategies that maximise population persistence in a given portion of the landscape (i.e., short dispersal) may also lead to the longest lags in expansion.

### Changing processes in the core and edge of the range

Predicting how metapopulations will change when in disequilibrium with the environment is made more challenging because key population processes and individual traits may vary from the core to the edge of the species range (Hargreaves and Eckert 2014; Morgan et al. 2019). We extended previous findings that post-dispersal mate-finding requirements led to a higher proportion of unmated females (Shaw and Kokko 2015) by revealing large spatial variation in mating success from the core to the edge of the range. We found a dramatic increase in the proportion of unmated females towards the range edge in our simulations, as well as clear spatial dependencies on the effect of dispersal decisions on female mating success. At the range edge, mate-searching caused a dramatic reduction in the proportion of unmated females. In core areas, dispersal decisions had little effect on female mating success, and the prevailing mating system (polygyny vs monogamy) was the more important factor.

Informed dispersal strategies can play an important role in mitigating the mate-finding Allee effects experienced at low densities (Gascoigne et al. 2009), but here we revealed how different combinations of behavioural traits may have very different effects on mating success in different parts of the species range. Species that adopt suites of behavioural traits most suited to range expansion will have a signficant advantage when exploiting newly emerging habitats under rapid environmental change. This may have important consequences for whole communities, and models suggest that where there is within-community variation in the capacity for range expansion, rapid environmental change has the potential to alter drastically both community assemblages and their associated interspecific interactions (Urban et al. 2012). It is well established that the order in which species colonise newly available habitat can have important consequences for community composition (Sutherland 1974; Connell and Slatyer 1977; Shulman et al. 1983) and, therefore, even small cross-species differences in the capacity to spread may lead to dramatic differences in eventual geographic distributions: rapid colonisers will escape competition and monopolise newly available environments, whereas those that lag behind risk being permanently excluded.

## Conclusion

Our findings highlight how even species that are able to persist as metapopulations in fragmented landscapes may experience long (multi generation) periods of disequilibrium following environmental change. While a subset of species may spread rapidly, others—likely those opting for monogamous mating strategies due to more constrained dispersal or a need for biparental care—may spread more slowly, despite relatively fast rates of reproduction. The knock-on effects of the lags experienced by individual species may be even greater lags before whole communities are able to shift in response to environmental change.

### Supplementary Information

Below is the link to the electronic supplementary material.

Supplementary file1 (PDF 279 KB)

## Data Availability

Links to full code for analysis and data are provided in the supplementary material. Data and code for analysis has been uploaded to Figshare and remains private. For the purpose of open access, the author has applied a Creative Commons Attribution (CC BY) licence to any Author Accepted Manuscript version arising from this submission. Full code for RangeShifter 2.0 software is available here: https://rangeshifter.github.io/portfolio/rangeshifter2.0/.
