# Supplementary material for: Mating system induced lags in rates of range expansion for different simulated mating systems and dispersal strategies: a modelling study
Source: Oecologia. 2024 Jan 3;204(1):119–32. doi: 10.1007/s00442-023-05492-w (PMC10830608; doi:10.1007/s00442-023-05492-w)
Supplement: Supplementary file 1 — Supplementary file1 (PDF 279 KB) [file 442_2023_5492_MOESM1_ESM.pdf]

Supplementary materials

Full code for analysis, data and parameter files are available on figshare:

Full code for analysis: https://figshare.com/s/92d82b81ce230c0e34ad

This is the complete code for the analysis. On running code, ensure that density-independent and density-dependent files are stored in different directories:

Landscape file: https://figshare.com/s/64841a07965604505909

The landscape file used by RangeShifter to initialise all subpopulations. Some parts of the analysis also make use of this file.

Data - Experiment 1: https://figshare.com/s/12d725a5f72e569b4b18

Files needed to carry out experiment 1 – metapopulation size and rate of range expansion under the three mating systems explored

Data - experiment 2 – density-independent: https://figshare.com/s/3e51324d285585f2d7d6

Files needed to carry out part of experiment 2 – rate of range expansion for density-independent settlement with or without mate-searching

Data - experiment 2 - density-dependent settlement: https://figshare.com/s/c812002c01c428c76bcb

Files needed to carry out part of experiment 2 – rate of range expansion for density-dependent settlement with or without mate-searching

Dispersal distances – one sex model/two sex model Density Dependent/Independent:

https://figshare.com/s/4c72f5b0610b46ab43dc

https://figshare.com/s/5ce9c4493cbc4f281c3c

https://figshare.com/s/dc93fd18e7ca00f1969e

Files needed to plot dispersal distances

Quasi-equilibrium occupancy in core

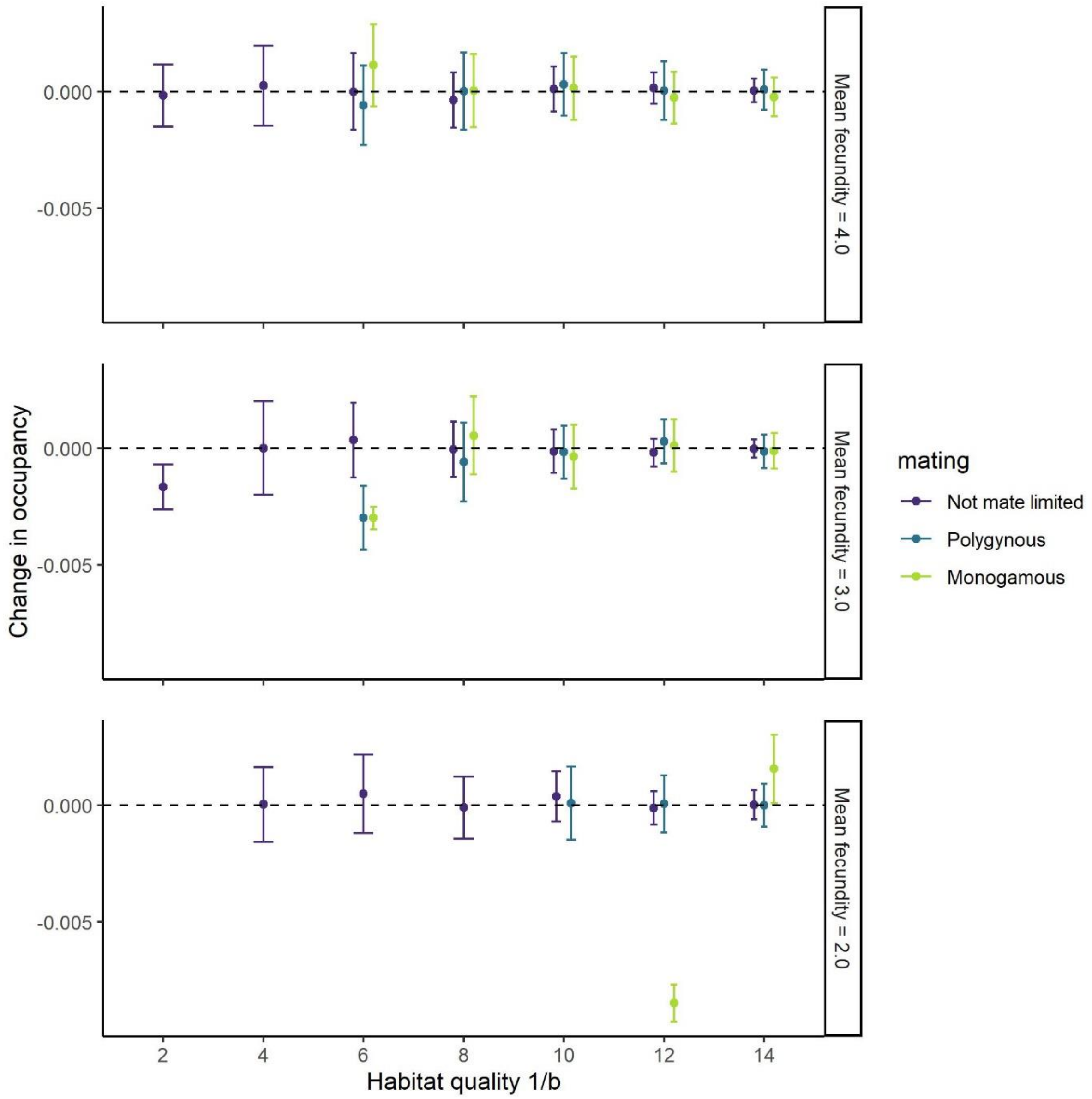

Figure S1. The effect of habitat quality on generational change in proportional occupancy in the first half of the landscape for simulations of three mating strategies for three values of fecundity. Only those simulations where species did not go extinct within 50 years are shown. Error bars show the standard error.

Example parameter files for different models

Not mate limited, no mate searching, density-independent settlement

```
RangeShifter 2.0  - 64 bit implementation
================    =====================

BATCH MODE      yes
REPLICATES      5
YEARS   50
REPRODUCTIVE SEASONS / YEAR   1
CELL-BASED MODEL
BOUNDARIES      reflective

LANDSCAPE:      imported map
TYPE:   habitat codes
FILE NAME:  (see batch file) C:\Permeability\RangeShifter\Water
voles\Repeat_RSv2\Female_only\Inputs\Landfile_5a.txt
No. HABITATS:  2
RESOLUTION (m):        100
DIMENSIONS:  X 20  Y 1000
AVAILABLE:   min.X 0 min.Y 0  max.X 19 max.Y 999

SPECIES DISTRIBUTION LOADED:  no

ENVIRONMENTAL GRADIENT:        no
ENVIRONMENTAL STOCHASTICITY:  no
LOCAL EXTINCTION PROBABILITY: 0.0

SPECIES' PARAMETERS.
REPRODUCTION:
TYPE: Asexual / Only female model
STAGE STRUCTURE:       yes
PROBABILITY OF REPRODUCING IN SUBSEQUENT SEASONS     1
No. OF REP. SEASONS BEFORE SUBSEQUENT REPRODUCTIONS  0
No. STAGES      2
MAX. AGE        2
MIN. AGES:
stage   0:      0       years
stage   1:      0       years
FECUNDITIES:
stage   0:      0
stage   1:      2
DEVELOPMENT PROB.:
stage   0:      1
stage   1:      0
SURVIVAL PROB.:
stage   0:      1
stage   1:      0.3
SCHEDULING OF SURVIVAL: Between reproductive events
DENSITY-DEPENDENCE IN FECUNDITY:      no
DENSITY-DEPENDENCE IN DEVELOPMENT:    no
DENSITY-DEPENDENCE IN SURVIVAL:              yes - coefficient: 1
not stage-dependent

HABITAT SPECIFIC 1/b:
Habitat 1:      1/b 0
Habitat 2:      1/b 2

DISPERSAL - EMIGRATION:       density-independent
SEX-DEPENDENT:   no
STAGE-DEPENDENT: yes
INDIVIDUAL VARIABILITY: no
stage 0:       EMIGRATION PROB.: 0.5
stage 1:       EMIGRATION PROB.: 0

DISPERSAL - TRANSFER:  SMS     costs:
        hab. 1  10
        hab. 2  1
PERCEPTUAL RANGE:        5
PERCEPTUAL RANGE METHOD: 2
```

```
DIRECTIONAL PERSISTENCE: 3
MEMORY SIZE: 1
GOAL TYPE:   0
INDIVIDUAL VARIABILITY: no
STRAIGHTEN PATH AFTER DECISION NOT TO SETTLE: yes
STEP MORTALITY:
constant 0.005

DISPERSAL - SETTLEMENT:
MIN. No. OF STEPS:      0
MAX. No. OF STEPS:      not applied
SEX-DEPENDENT:   no
STAGE-DEPENDENT: no
SETTLE IF: find a suitable cell/patch (not the natal one)
MAX. No. OF STEPS/YEAR:        not applied

GENETICS:
No. of variable traits:  0

INITIALISATION CONDITIONS:
From initial individuals file: C:\Permeability\RangeShifter\Water
voles\Repeat_RSv2\Female_only\Inputs\init_fem_half_50.L2.1.txt

OUTPUTS:
Range - every 1 year
Occupancy - every 1 year
Populations - every 1 year
SAVE MAPS: no
SAVE TRAITS MAPS: no
SMS HEAT MAPS: no
```

Polygynous mating, no mate searching, density-independent settlement

```
RangeShifter 2.0  - 64 bit implementation
================    =====================

BATCH MODE     yes
REPLICATES     5
YEARS   50
REPRODUCTIVE SEASONS / YEAR   1
CELL-BASED MODEL
BOUNDARIES     reflective

LANDSCAPE:     imported map
TYPE:   habitat codes
FILE NAME:  (see batch file) C:\Water_voles\Two_sex_dens_indep\Inputs\Landfile_5.txt
No. HABITATS:  2
RESOLUTION (m):        100
DIMENSIONS:  X 20  Y 1000
AVAILABLE:   min.X 0 min.Y 0  max.X 19 max.Y 999

SPECIES DISTRIBUTION LOADED:  no

ENVIRONMENTAL GRADIENT:        no
ENVIRONMENTAL STOCHASTICITY:  no
LOCAL EXTINCTION PROBABILITY: 0.0

SPECIES' PARAMETERS.
REPRODUCTION:
TYPE: Sexual model (explicit mating system)
PROP. of MALES 0.5
MAX. HAREM SIZE (h)    100
STAGE STRUCTURE:       yes
PROBABILITY OF REPRODUCING IN SUBSEQUENT SEASONS    1
No. OF REP. SEASONS BEFORE SUBSEQUENT REPRODUCTIONS  0
No. STAGES     2
MAX. AGE       2
MIN. AGES:
males 0:       0 years;       females 0:     0 years
males 1:       0 years;       females 1:     0 years
FECUNDITIES:
```

```
males   0:     0
females 0:     0
males   1:     1
females 1:     4
DEVELOPMENT PROB.:
males   0:     1
females 0:     1
males   1:     0
females 1:     0
SURVIVAL PROB.:
males   0:     1
females 0:     1
males   1:     0.3
females 1:     0.3
SCHEDULING OF SURVIVAL: Between reproductive events
DENSITY-DEPENDENCE IN FECUNDITY:      no
DENSITY-DEPENDENCE IN DEVELOPMENT:    no
DENSITY-DEPENDENCE IN SURVIVAL:               yes - coefficient: 1
STAGE'S WEIGHTS:
stage 0 males  :       0      0      0      0
stage 0 females:       1      1      1      1
stage 1 males  :       0      0      0      0
stage 1 females:       1      1      1      1

HABITAT SPECIFIC 1/b:
Habitat 1:     1/b 0
Habitat 2:     1/b 2

DISPERSAL - EMIGRATION:       density-independent
SEX-DEPENDENT:   no
STAGE-DEPENDENT: yes
INDIVIDUAL VARIABILITY: no
stage 0:       EMIGRATION PROB.: 0.5
stage 1:       EMIGRATION PROB.: 0

DISPERSAL - TRANSFER:  SMS     costs:
       hab. 1  10
       hab. 2  1
PERCEPTUAL RANGE:        5
PERCEPTUAL RANGE METHOD: 2
DIRECTIONAL PERSISTENCE: 3
MEMORY SIZE: 1
GOAL TYPE:   0
INDIVIDUAL VARIABILITY: no
STRAIGHTEN PATH AFTER DECISION NOT TO SETTLE: yes
STEP MORTALITY:
constant 0.005

DISPERSAL - SETTLEMENT:
MIN. No. OF STEPS:      0
MAX. No. OF STEPS:      not applied
SEX-DEPENDENT:   no
STAGE-DEPENDENT: no
SETTLE IF: find a suitable cell/patch (not the natal one)
MAX. No. OF STEPS/YEAR:        not applied

GENETICS:
No. of variable traits:  0

INITIALISATION CONDITIONS:
From initial individuals file:
C:\Water_voles\Two_sex_dens_indep\Inputs\init_2sex_half_50.L2.1.txt

OUTPUTS:
Range - every 1 year
Occupancy - every 1 year
Populations - every 1 year
SAVE MAPS: no
SAVE TRAITS MAPS: no
SMS HEAT MAPS: no
```

Monogamous mating, no mate searching, density-independent settlement

```
RangeShifter 2.0  - 64 bit implementation
================    =====================

BATCH MODE     yes
REPLICATES     5
YEARS   50
REPRODUCTIVE SEASONS / YEAR   1
CELL-BASED MODEL
BOUNDARIES     reflective

LANDSCAPE:     imported map
TYPE:   habitat codes
FILE NAME:  (see batch file) C:\Water_voles\Two_sex_dens_indep\Inputs\Landfile_5.txt
No. HABITATS:  2
RESOLUTION (m):        100
DIMENSIONS:  X 20  Y 1000
AVAILABLE:   min.X 0 min.Y 0  max.X 19 max.Y 999

SPECIES DISTRIBUTION LOADED:  no

ENVIRONMENTAL GRADIENT:        no
ENVIRONMENTAL STOCHASTICITY:  no
LOCAL EXTINCTION PROBABILITY: 0.0

SPECIES' PARAMETERS.
REPRODUCTION:
TYPE: Sexual model (explicit mating system)
PROP. of MALES 0.5
MAX. HAREM SIZE (h)    1
STAGE STRUCTURE:       yes
PROBABILITY OF REPRODUCING IN SUBSEQUENT SEASONS     1
No. OF REP. SEASONS BEFORE SUBSEQUENT REPRODUCTIONS  0
No. STAGES     2
MAX. AGE       2
MIN. AGES:
males 0:       0 years;       females 0:     0 years
males 1:       0 years;       females 1:     0 years
FECUNDITIES:
males   0:     0
females 0:     0
males   1:     1
females 1:     4
DEVELOPMENT PROB.:
males   0:     1
females 0:     1
males   1:     0
females 1:     0
SURVIVAL PROB.:
males   0:     1
females 0:     1
males   1:     0.3
females 1:     0.3
SCHEDULING OF SURVIVAL: Between reproductive events
DENSITY-DEPENDENCE IN FECUNDITY:      no
DENSITY-DEPENDENCE IN DEVELOPMENT:    no
DENSITY-DEPENDENCE IN SURVIVAL:               yes - coefficient: 1
STAGE'S WEIGHTS:
stage 0 males  :       0      0      0      0
stage 0 females:       1      1      1      1
stage 1 males  :       0      0      0      0
stage 1 females:       1      1      1      1

HABITAT SPECIFIC 1/b:
Habitat 1:     1/b 0
Habitat 2:     1/b 2

DISPERSAL - EMIGRATION:       density-independent
SEX-DEPENDENT:   no
STAGE-DEPENDENT: yes
```

```
INDIVIDUAL VARIABILITY: no
stage 0:       EMIGRATION PROB.: 0.5
stage 1:       EMIGRATION PROB.: 0

DISPERSAL - TRANSFER:  SMS     costs:
        hab. 1  10
        hab. 2  1
PERCEPTUAL RANGE:        5
PERCEPTUAL RANGE METHOD: 2
DIRECTIONAL PERSISTENCE: 3
MEMORY SIZE: 1
GOAL TYPE:   0
INDIVIDUAL VARIABILITY: no
STRAIGHTEN PATH AFTER DECISION NOT TO SETTLE: yes
STEP MORTALITY:
constant 0.005

DISPERSAL - SETTLEMENT:
MIN. No. OF STEPS:      0
MAX. No. OF STEPS:      not applied
SEX-DEPENDENT:   no
STAGE-DEPENDENT: no
SETTLE IF: find a suitable cell/patch (not the natal one)
MAX. No. OF STEPS/YEAR:        not applied

GENETICS:
No. of variable traits:  0

INITIALISATION CONDITIONS:
From initial individuals file:
C:\Water_voles\Two_sex_dens_indep\Inputs\init_2sex_half_50.L2.1.txt

OUTPUTS:
Range - every 1 year
Occupancy - every 1 year
Populations - every 1 year
SAVE MAPS: no
SAVE TRAITS MAPS: no
SMS HEAT MAPS: no
```

Polygynous mating, mate searching, density-dependent settlement

```
RangeShifter 2.0  - 64 bit implementation
================    =====================

BATCH MODE     yes
REPLICATES     5
YEARS   50
REPRODUCTIVE SEASONS / YEAR   1
CELL-BASED MODEL
BOUNDARIES     reflective

LANDSCAPE:     imported map
TYPE:   habitat codes
FILE NAME:  (see batch file) C:\Water_voles\Two_sex_dens_dep\Inputs\Landfile_5.txt
No. HABITATS:  2
RESOLUTION (m):        100
DIMENSIONS:  X 20  Y 1000
AVAILABLE:   min.X 0 min.Y 0  max.X 19 max.Y 999

SPECIES DISTRIBUTION LOADED:  no

ENVIRONMENTAL GRADIENT:        no
ENVIRONMENTAL STOCHASTICITY:  no
LOCAL EXTINCTION PROBABILITY: 0.0

SPECIES' PARAMETERS.
REPRODUCTION:
TYPE: Sexual model (explicit mating system)
PROP. of MALES 0.5
MAX. HAREM SIZE (h)    100
```

```
STAGE STRUCTURE:       yes
PROBABILITY OF REPRODUCING IN SUBSEQUENT SEASONS     1
No. OF REP. SEASONS BEFORE SUBSEQUENT REPRODUCTIONS  0
No. STAGES      2
MAX. AGE        2
MIN. AGES:
males 0:        0 years;        females 0:      0 years
males 1:        0 years;        females 1:      0 years
FECUNDITIES:
males   0:      0
females 0:      0
males   1:      1
females 1:      6
DEVELOPMENT PROB.:
males   0:      1
females 0:      1
males   1:      0
females 1:      0
SURVIVAL PROB.:
males   0:      1
females 0:      1
males   1:      0.3
females 1:      0.3
SCHEDULING OF SURVIVAL: Between reproductive events
DENSITY-DEPENDENCE IN FECUNDITY:      no
DENSITY-DEPENDENCE IN DEVELOPMENT:    no
DENSITY-DEPENDENCE IN SURVIVAL:               yes - coefficient: 1
STAGE'S WEIGHTS:
stage 0 males  :       0       0       0       0
stage 0 females:       1       1       1       1
stage 1 males  :       0       0       0       0
stage 1 females:       1       1       1       1

HABITAT SPECIFIC 1/b:
Habitat 1:      1/b 0
Habitat 2:      1/b 6

DISPERSAL - EMIGRATION:        density-independent
SEX-DEPENDENT:   no
STAGE-DEPENDENT: yes
INDIVIDUAL VARIABILITY: no
stage 0:        EMIGRATION PROB.: 0.5
stage 1:        EMIGRATION PROB.: 0

DISPERSAL - TRANSFER:  SMS     costs:
        hab. 1  10
        hab. 2  1
PERCEPTUAL RANGE:         5
PERCEPTUAL RANGE METHOD: 2
DIRECTIONAL PERSISTENCE: 3
MEMORY SIZE: 1
GOAL TYPE:   0
INDIVIDUAL VARIABILITY: no
STRAIGHTEN PATH AFTER DECISION NOT TO SETTLE: yes
STEP MORTALITY:
constant 0.005

DISPERSAL - SETTLEMENT:
MIN. No. OF STEPS:      0
MAX. No. OF STEPS:      not applied
SEX-DEPENDENT:   no
STAGE-DEPENDENT: no
SETTLE IF: find a suitable cell/patch + density dependence + mating requirements
S0: 1  AlphaS: -10  BetaS: 0.75
MAX. No. OF STEPS/YEAR:        not applied

GENETICS:
No. of variable traits:  0

INITIALISATION CONDITIONS:
```

From initial individuals file:
C:\Water_voles\Two_sex_dens_dep\Inputs\init_2sex_half_50.L2.1.txt

OUTPUTS:
Range - every 1 year
Occupancy - every 1 year
Populations - every 1 year
SAVE MAPS: no
SAVE TRAITS MAPS: no
SMS HEAT MAPS: no
